# Supplementary material for: Assessment of a Loop-Mediated Isothermal Amplification (LAMP) Assay for the Rapid Detection of Pathogenic Bacteria from Respiratory Samples in Patients with Hospital-Acquired Pneumonia
Source: Microorganisms. 2020 Jan 11;8(1):103. doi: 10.3390/microorganisms8010103 (PMC7022425; doi:10.3390/microorganisms8010103)
Supplement: Supplementary file 1 [file microorganisms-08-00103-s001.pdf]

Supplementary table. LAMP results from polymicrobial BAS/EA samples according to the result obtained by culture.

**Type of error**	**Culture**	**LAMP**
Major	<1000 ufc of *S. aureus*<br><1000 ufc of *P. aeruginosa*	Negative
Major	>10000 ufc of *S. aureus*<br>2000000 ufc of *P. aeruginosa*	*S. aureus* not detected<br>*P. aeruginosa* and *E. coli* detected
Major	300000 ufc of *A. baumannii*<br>300000 ufc of *E. coli*	*A. baumannii* not detected<br>*E. coli*, *P. aeruginosa* and *S. maltophilia* detected
Minor	40000 ufc of *P. aeruginosa*<br>100000 ufc of *E. coli*	*E. coli*, *P. aeruginosa* and *S. maltophilia* detected
Minor	500000 ufc of *S. aureus*<br>3000000 ufc of *A. baumannii*	*S. aureus, A. baumannii* and *K. pneumoniae* detected
Minor	900000 ufc of *P. aeruginosa*<br>80000 ufc of *K. pneumoniae*	*P. aeruginosa*, *K. pneumoniae, S. aureus* and *S. maltophilia* detected
Minor	1000 ufc of *S. aureus*<br>60000 ufc of *S. maltophilia*	*S. aureus, S. maltophilia* and *E. coli* detected
